# Finding a Fox: An Evaluation of Survey Methods to Estimate Abundance of a Small Desert Carnivore

**DOI:** 10.1371/journal.pone.0105873

**Published:** 2014-08-22

**Authors:** Steven J. Dempsey, Eric M. Gese, Bryan M. Kluever

**Affiliations:** 1 Department of Wildland Resources, Utah State University, Logan, Utah, United States of America; 2 United States Department of Agriculture, Animal and Plant Health Inspection Service, Wildlife Services, National Wildlife Research Center, Department of Wildland Resources, Utah State University, Logan, Utah, United States of America; Smithsonian Conservation Biology Institute, United States of America

## Abstract

The status of many carnivore species is a growing concern for wildlife agencies, conservation organizations, and the general public. Historically, kit foxes (*Vulpes macrotis*) were classified as abundant and distributed in the desert and semi-arid regions of southwestern North America, but is now considered rare throughout its range. Survey methods have been evaluated for kit foxes, but often in populations where abundance is high and there is little consensus on which technique is best to monitor abundance. We conducted a 2-year study to evaluate four survey methods (scat deposition surveys, scent station surveys, spotlight survey, and trapping) for detecting kit foxes and measuring fox abundance. We determined the probability of detection for each method, and examined the correlation between the relative abundance as estimated by each survey method and the known minimum kit fox abundance as determined by radio-collared animals. All surveys were conducted on 15 5-km transects during the 3 biological seasons of the kit fox. Scat deposition surveys had both the highest detection probabilities (p = 0.88) and were most closely related to minimum known fox abundance (*r^2^* = 0.50, *P* = 0.001). The next best method for kit fox detection was the scent station survey (p = 0.73), which had the second highest correlation to fox abundance (*r^2^* = 0.46, *P*<0.001). For detecting kit foxes in a low density population we suggest using scat deposition transects during the breeding season. Scat deposition surveys have low costs, resilience to weather, low labor requirements, and pose no risk to the study animals. The breeding season was ideal for monitoring kit fox population size, as detections consisted of the resident population and had the highest detection probabilities. Using appropriate monitoring techniques will be critical for future conservation actions for this rare desert carnivore.

## Introduction

Populations of large and small carnivores are threatened or imperiled throughout the world [1]. With increasing human populations and subsequent habitat loss and fragmentation, declines in natural prey, increased human persecution and illegal poaching, many carnivore species have declined in number and now occupy a fragment of their former range. Paramount to species management and conservation is knowledge about the status and distribution of many carnivore species. A question often facing wildlife agencies and conservation groups is how many animals are there and what is the population trend? However, many carnivore species are difficult to survey due to their low densities, are generally nocturnal and elusive, and wary of humans [2]–[6].

Kit foxes (*Vulpes macrotis*) are a slim, small canid (1–3 kg body mass) with ears that are relatively larger than those of other North American canids, is considered to be monestrus and socially monogamous, and is a dietary generalist feeding on rodents, insects, lagomorphs, ground-nesting birds, and reptiles [7]. Historically, kit foxes were once abundant and distributed throughout the desert and semi-arid regions of southwestern North America, ranging from Idaho to central Mexico [7]. Their range-wide decline has warranted the kit fox to be state-listed as endangered in Colorado, threatened in California and Oregon, and designated as a state sensitive species in Idaho and Utah [8]. However, a comprehensive study of kit fox abundance across its range is lacking, with the majority of studies focused on the endangered subspecies, the San Joaquin kit fox (*V. macrotis mutica*). In Utah, where kit foxes were once considered the most abundant carnivore in the west desert [9], [10], the kit fox has been in steep decline over the past decade [2], [11], [12].

Current methods used for surveying kit foxes and their close relative the swift fox (*V. velox*), include capture-recapture [2], [13]–[17], spotlight surveys [2], [13]–[15], [18], scent station surveys [2], [13]–[15], scat deposition transects with and without scat detection dogs [2], [14], [15], [17], [19], track counts [14], activity index [15], and howling response [14]. Generally these methods have been evaluated in study areas with a relatively high fox density. How well these methods will perform for monitoring fox abundance in a low-density, widely dispersed kit fox population is unknown. We tested 4 survey methods (scat deposition, scent station, spotlight, trapping) on the U.S. Army Dugway Proving Ground (DPG), Utah, with the primary objectives to (1) determine detection probabilities for each method, and (2) evaluate how well the indices of relative abundance for each survey method correlate with known kit fox abundance as determined from available radio-collared animals. The kit fox population in the west desert of Utah is considered low density, declining in abundance, and widely dispersed [11], [20], [21].

## Methods

### Ethics Statement

Fieldwork was approved and sanctioned by the United States Department of Agriculture's National Wildlife Research Center and the United States Army's Dugway Proving Ground. Permission to access land on the Dugway Proving Ground was obtained from the United States Army; permission to access Bureau of Land Management property was obtained from the Bureau of Land Management.

Capture and handling protocols were reviewed and approved by the Institutional Animal Care and Use Committees (IACUC) at the United States Department of Agriculture's National Wildlife Research Center (QA-1734) and Utah State University (#1438). Permits to capture, handle, and radio-collar kit foxes were obtained from the Utah Division of Wildlife Resources (COR #4COLL8322). Data is archived and available from the United States Department of Agriculture's National Wildlife Research Center (QA-1734).

### Study Area

We conducted this study on 879 km^2^ of the eastern portion of the DPG and the adjoining land managed by the Bureau of Land Management, located approximately 128 km southwest of Salt Lake City, in Tooele County, Utah. Elevations ranged from 1302 m to 2137 m. The study site was in the Great Basin Desert and was characterized as a cold desert. Winters were cold, and summers were hot and dry with the majority of precipitation occurring in the spring [11]. The study area consisted of predominately flat playa punctuated with steep mountain ranges. We classified the landscape into 7 vegetation communities: chenopod, greasewood, pickle weed, grassland, stable dune, shrub-steppe, and urban; see [20], [21] for a detailed description of vegetation communities.

### Animal Capture and Handling

Beginning in December 2009, we captured kit foxes via transect trapping [15] and at known den sites [20], [22], using box traps (25×25×80 cm; Model 107; Tomahawk Live Trap LLC, Hazelhurst, Wisconsin) baited with hot dogs. Trapping transects were distributed to provide maximum coverage of the area and allow for increased likelihood of capturing most of the kit foxes occupying the study area [11], [15], [20]. We deployed traps in the evening and checked them early morning each day. We coaxed captured foxes into a canvas bag placed at the edge of the trap, then restrained by personnel wearing thick leather gloves [22]. We weighed, sexed, ear tagged, and fitted each fox with a 30–50 g radio-collar (Model M1930; Advanced Telemetry Systems, Isanti, Minnesota). Collars included a mortality sensor that activated after 6 hours of non-motion and weighed <5% of body mass [15], [23], [24]. We handled all foxes without the use of immobilizing drugs and released them at the capture site.

### Radio-telemetry and Home Range Determination

We collected animal locations >3 times per week using a portable receiver (Model R1000; Communications Specialists, Inc., Orange, California) and a handheld 3-element Yagi antenna. We triangulated an animal's location using ≥2 compass bearings, each >20° but <160° apart, for each animal within 20 minutes [11], [20]. We then calculated their location using program Locate III (Pacer Computing, Tatamagouche, Nova Scotia). For each week, we temporally distributed telemetry sampling by collecting two crepuscular (hunting) locations and one den (resting) location. To reduce auto-correlation and retain temporal independence between locations, we separated each crepuscular sample by >12 hours and a difference of >2 hours in the time of day of each location [25]–[27]. We collected one weekly den location for each animal by homing in on the signal during daylight hours. We attempted to locate each fox ≥3 times weekly in order to obtain 40 locations for each fox for each biological season as the minimum number of locations needed to adequately describe the home range of a fox [27].

To determine space use of kit foxes, we created seasonal home ranges for all kit foxes with ≥30 locations within the season [27], [28]. We defined the biological seasons based on the behavior and energetic needs of kit foxes: breeding 15 December – 14 April, pup-rearing 15 April – 14 August and dispersal 15 August – 14 December [10], [24], [29]. We created home range polygons using the Home-Range Analysis and Estimation (HoRAE) toolbox for the Open Jump geographic information system [30]. We created 95% point kernel density estimates (KDE) using a fixed kernel (standard sextante biweight) and the ad hoc method [31], [32] for determination of the smoothing parameter *h* (e.g., *h_ref_*, 90%*h_ref_*, 80%*h_ref_*, 70%*h_ref_*, etc.). This method was designed to prevent over/under-smoothing and selection of the tightest fitting contiguous home range polygon before developing discrete patches [32]–[34]. We then loaded these polygons into ArcMap 10.0 (Environmental Systems Research Institute Inc., Redlands, CA) to calculate kit fox home range size.

### Surveys

**Figure 1:**
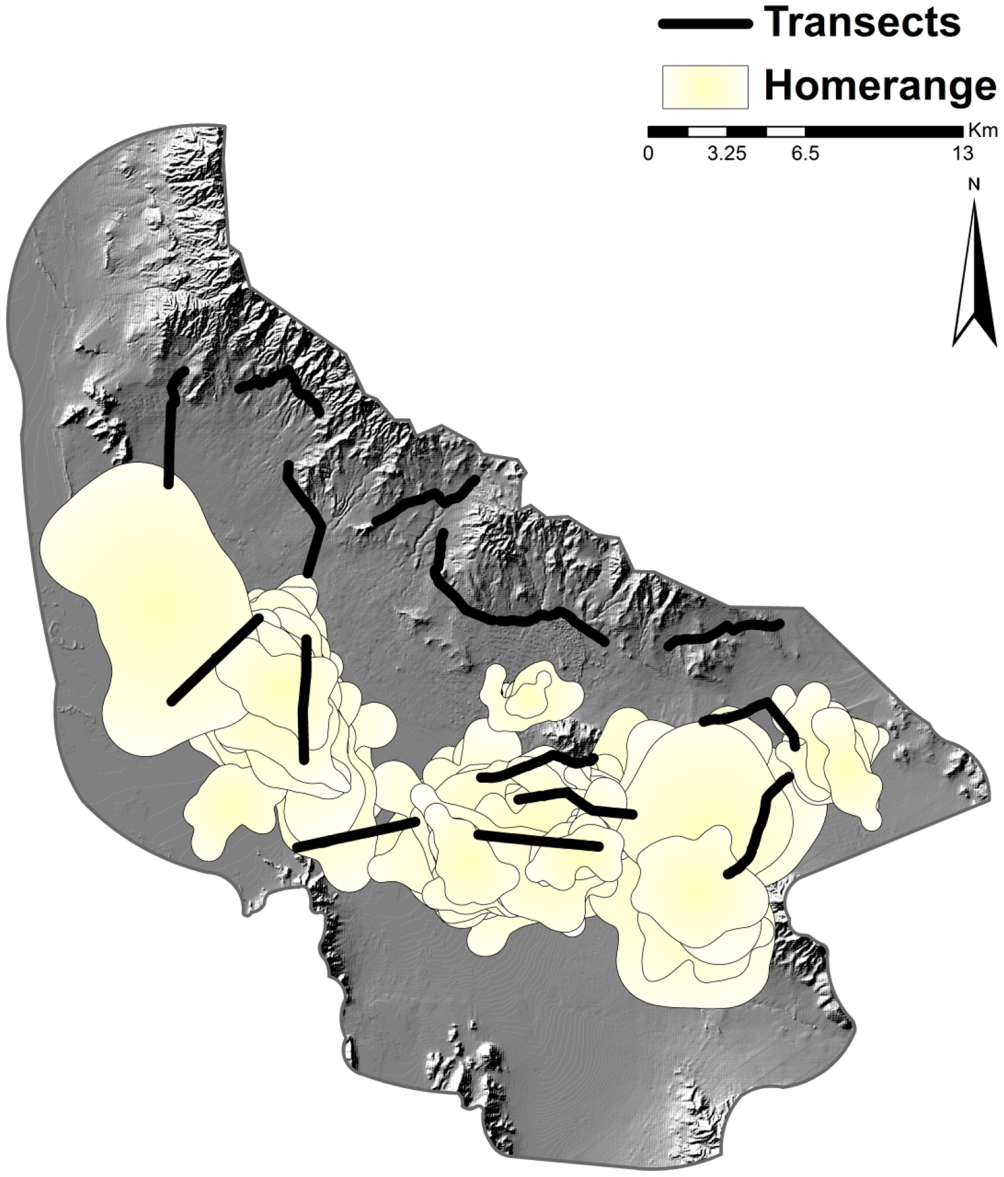
Transects and all kit fox home ranges created from telemetry locations, Dugway Proving Ground, Utah, 2010–2012; two transects were conducted along the same road and appear to be connected, but the end points were independent.

From March 2010 to April 2012, we attempted to conduct four different surveys (scat deposition, scent station, spotlight, and trapping) during each of the three biological kit fox seasons (breeding, pup-rearing, and dispersal) for two years. The surveys were initiated after the trapping and collaring effort due to our needing to know the number of foxes available along the transects prior to a survey period; thus the surveys did not begin until the pup rearing season of 2010. We conducted each survey along 15 5-km established transects (Figure 1). We distributed transects randomly along available roads with the constraints of being as linear as possible and having year-round access (limitations included military closures and low lying seasonally inundated greasewood areas). We attempted to conduct 4 consecutive nights of scent-station, spotlighting, and trapping surveys during each of the biological seasons along all 15 transects. Each survey was conducted separately along the transects; i.e., surveys were not conducted simultaneously on the same transect but one type of survey was conducted simultaneously over multiple transects. Due to concerns of overheating and the demands of natal care of female foxes, we did not conduct the trapping survey during the pup-rearing season. High winds, snowfall, and melting and freezing cycles limited our ability to complete some surveys during the winter months; scent stations were the most affected by weather. Most notably, of the attempted 660 survey nights possible during the breeding season of 2011, only 462 (70%) scent stations were operable.

Initially designed for lagomorph counts, we conducted spotlight surveys for 3 consecutive nights during the pup rearing season of 2010 and the dispersal season of 2010. We modified our methodology and performed the spotlight surveys over 4 nights the remainder of the study. Two methods (scent stations and trapping surveys) were point sampling techniques with 11 discrete locations for detection. The remaining 2 techniques (scat deposition and spotlight surveys) allowed for detection along the entire length of a transect. Additionally, 2 techniques (scat deposition and scent stations) allowed for an individual animal to be detected multiple times along a transect, while during trapping or spotlight surveys an individual may only be detected once.

#### Scat deposition survey

We conducted scat deposition surveys by initially walking the transect to clear any scat from the road surface, then returning approximately 14 days later to walk and count the number of scats deposited [13], [15]. Following recommendations [15], [35], we walked each transect in both directions to reduce missed detections of scats. We recorded the scat location and type (species) on a handheld GPS unit, and collected the scat. This provided a count of the total number of scats per transect (surveys were a constant 5-km length and 14-day duration) as a measure of relative abundance [3].

#### Scent station survey

We placed scent stations at 0.5 km intervals on alternating sides along each 5 km transect [13], [15]. A scent station consisted of a cleared 1-m circle of lightly sifted sand [36] with a Scented Predator Survey Disk (SPSD; United States Department of Agriculture's Pocatello Supply Depot, Pocatello, Idaho) with Fatty Acid Scent (FAS) placed in the center. The SPSD with FAS was recommended for “ease of use, attractiveness to kit fox, and their low cost” [2]. FAS saturated SPSD's are preferred over the use of liquid lures because they allow for control of a consistent attractiveness between batches [37]. We checked *s*tations each morning for tracks of kit foxes, coyotes (*Canis latrans*), bobcats (*Lynx rufus*), leporids, small mammals, and other potential prey species. We then resifted each *s*tation and replaced the SPSD. To help maintain consistent attractiveness, we removed SPSD's from use once they were noticeably deteriorated, broken, or after a full season of use. We resampled inoperable station nights (due to inclement weather) for an additional 1–2 days in an attempt to complete the 4 nights of surveying. If transects remained inoperable after the additional days, we ceased the survey along that transect and results for that transect were not used in subsequent analysis. This survey provided a proportion of visited scent stations (i.e., total number of visits or detections divided by the number of operable stations) as a measure of relative abundance [3].

#### Spotlight survey

While driving a vehicle along the transect route at approximately 10–15 km/hr, 2 observers scanned their respective side of the road with a 3 million candlepower spotlight [13], [15], [18]. Once an animal was sighted, we stopped the vehicle and the species was identified. We recorded the species, location, distance, and bearing to the animal for kit foxes, coyotes, bobcats, and leporids. The survey provided a count of the total number of foxes detected divided by the number of nights surveyed as a measure of relative abundance [3].

#### Trapping survey

We conducted a trapping survey with box traps placed at 0.5 km intervals along each 5 km transect [15]. We baited traps with half of a hot dog, wired down towards the rear of the trap. We partially covered each trap with vegetation to deter a kit fox from digging under the trap for the bait. We checked traps daily, and re-baited them after two days or when a significant portion of the bait had deteriorated or had been eaten by small mammals. We deployed traps in the evening and closed them during the day to limit the amount of exposure to the animals [2]. We processed animals captured in this survey following the handling protocol previously described. We restricted trapping until late in the pup-rearing season to allow the foxes to mature enough to permit radio-collaring (i.e., they were old enough to be within our <5% body mass requirement for radio-collaring). This survey provided an index of foxes captured divided by the number of operable trap nights as a measure of relative abundance [3].

#### Detection probability

For each biological season we computed detection probabilities of each survey method with the occupancy estimator in Program MARK [38] that accommodates covariate information and missed observations [39]. To account for a measure of space use, we buffered each transect by one-third of the average radius of kit fox home range during each season [15]. A fox was considered available for detection if it was alive during the survey dates and it had locations within the transect buffer during that biological season. We fit models using 4 encounter occasions of 4 groups of survey methods (scat deposition, scent station, spotlight, and trapping), along with 3 covariates (survey year, number of radio-marked foxes available for detection, fox presence or absence). Fox presence or absence was binary and determined as presence if ≥1 fox was available for detection based on the criteria above. All possible models were examined and we selected the best model by AIC ranking [40].

### Correlation between Survey Indices and Fox Abundance

In addition to determining detection probabilities for each survey method, we also examined the correlation between the index of relative abundance for each survey method and the minimum number of known kit foxes along each transect (i.e., minimum abundance), similar to the evaluation conducted for swift foxes [15]. As described above, we determined the minimum number of known foxes along a transect by buffering each transect by one-third of the average radius of kit fox home range during each season [15]. A fox was considered available for sampling along that transect if it was alive during the survey dates and it had locations within the transect buffer during that biological season.

## Results

### Capture and Telemetry

From December 2009 to April 2012, we accumulated 6,221 trap nights and captured 45 (26 females, 19 males) foxes across the study area 106 times. During the study we obtained 4,498 fox locations (1,487 in breeding, 1,464 in dispersal, 1,547 in pup-rearing) allowing for the calculation of 66 seasonal home ranges (21 in breeding, 24 in dispersal, and 21 in pup rearing) (Figure 1). However, due to mid-season dispersal events, 2 foxes with >30 locations were not included in home range determinations.

### Home Range Estimation

We found seasonal 95% KDE home range sizes for kit foxes averaged 20.5 km^2^ (*n* = 64, *SD* = 15.1). For both years combined, average home range size of kit foxes during the dispersal season was 23.3 km^2^ (*n* = 23, *SD* = 16.1), followed by the breeding season (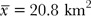, *n* = 20, *SD* = 17.8) and pup-rearing (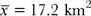, *n* = 21, *SD* = 9.4). These home range sizes (Table 1) were then used to buffer the transects to determine the known number of kit foxes available for each survey. The number of foxes available for detection along transects varied by survey type, season, and year, from a maximum of 9 foxes available along one transect during the dispersal season of 2010 to 5 transects on which there never was a known fox present during any season or year. Although individual transects may have not had known foxes present along them, there were always known foxes available for detection along some proportion of transects.

**Table 1:** Mean home range size (km^2^) for kit foxes, range of home range size, and number of foxes monitored for each biological season and year, Dugway Proving Ground, Utah, 2010–2012.

Season	Mean home range size (km^2^)	Range (km^2^)	n foxes
Pup-rearing 2010	18.8	1.7–28.0	12
Dispersal 2010	26.1	3.1–80.1	14
Breeding 2011	20.6	2.2–66.1	14
Pup-rearing 2011	15.0	2.4–38.9	9
Dispersal 2011	18.8	7.9–47.8	9
Breeding 2012	21.2	6.6–71.6	6

### Surveys

#### Detection probability

Detection probabilities were calculated for each transect to determine which survey method was best at detecting fox presence while controlling for differences in occupancy rates. For all biological seasons, the best model for detection probability (p) included differences across survey type (i.e., group) and the fox presence covariate. The corresponding best model for occupancy (Ψ) was constant across groups and the number of foxes available. This model fitted the expectation that each survey method would have a different p given the presence or absence of a fox available to be detected. Additionally, by holding Ψ constant and including a covariate for the minimum number of foxes available, we were able to include a known minimum number of foxes as determined through the space use information.

#### Scat deposition

**Figure 2:**
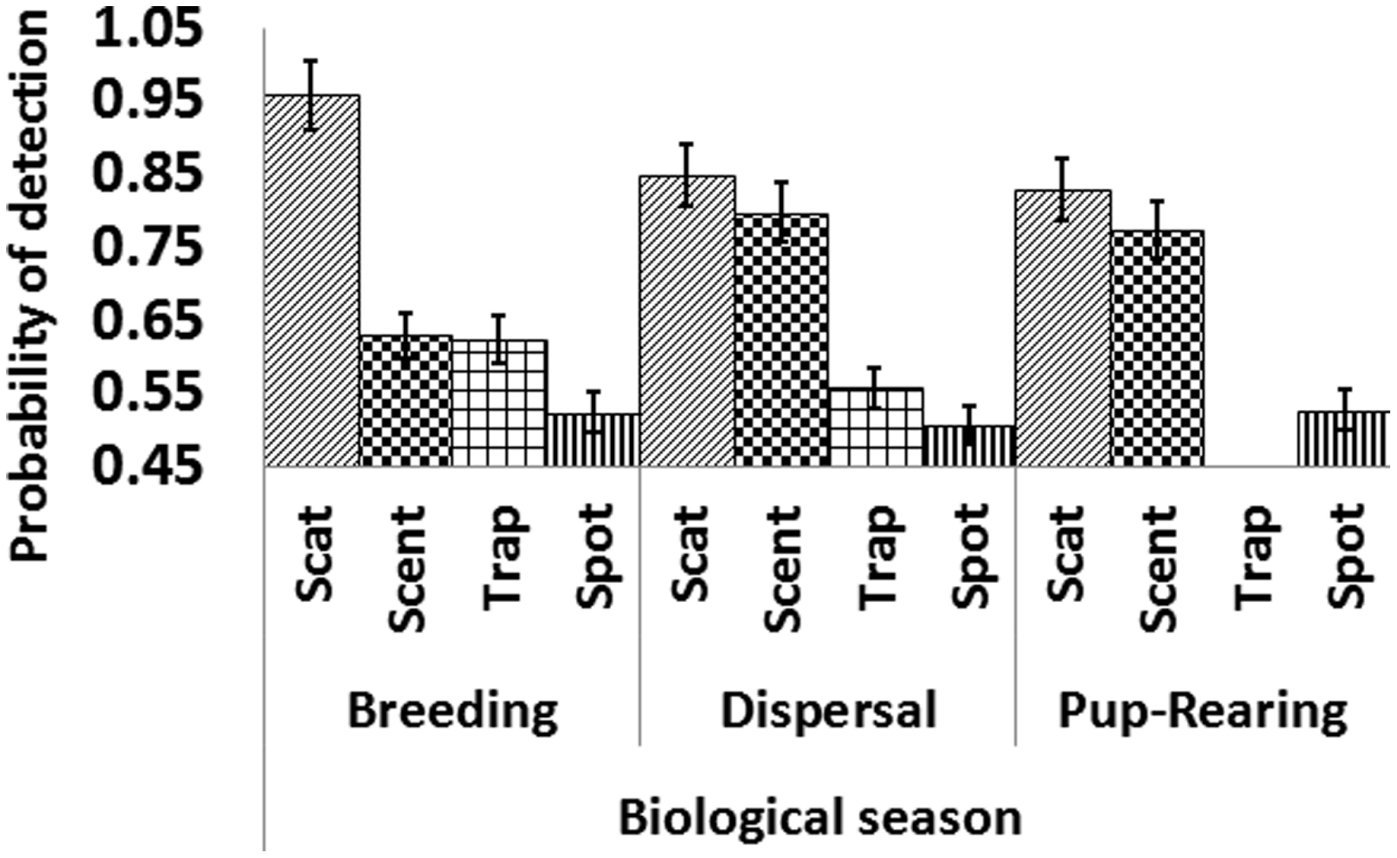
Probability of detection for scat deposition (Scat), scent station (Scent), trapping (Trap), and spotlight (Spot) surveys during 3 biological seasons for kit foxes on the Dugway Proving Ground, Utah, 2010–2012. Standard error bars included for each method.

**Figure 3:**
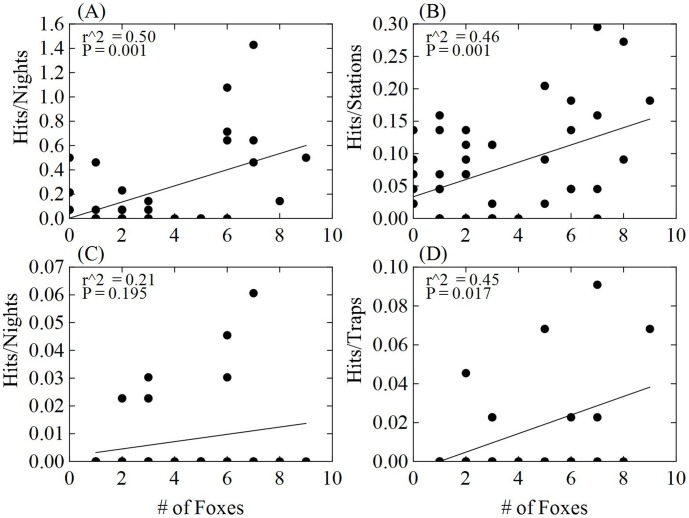
Relationship between the minimum number of known available foxes along the transects and indices of relative abundance for (A) scat deposition transects, (B) scent station surveys, (C) spotlight counts, and (D) trapping index, Dugway Proving Ground, Utah, 2010–2012.

We conducted 75 scat deposition surveys with 136 scat detections. Scat deposition produced the most detections (29) along an individual transect. Scat deposition surveys consistently had the highest detection probabilities (p = 0.88; Figure 2). Scat deposition surveys had the highest correlation (based on r^2^ value) with kit fox abundance (r^2^ = 0.50, P = 0.001). The correlation between scat detection rate and known fox abundance was linear and positive (Figure 3A).

#### Scent stations

Even with logistical difficulties due to weather, scent stations had the second highest detection probabilities (p = 0.73; Figure 2). Over the 3,718 operable station nights, we collected 159 fox detections. Scent stations had the second highest correlation with fox abundance which was linear and positive (r^2^ = 0.46, P<0.001; Figure 3B).

#### Spotlight surveys

The spotlight survey was the only method that did not detect fox presence during a complete biological season. During the dispersal season of 2011, the spotlight survey produced 0 detections although 18 radio-collared foxes were known to be available along the 15 transects. We completed 327 spotlight survey nights with 15 detections. Spotlight surveys had the lowest number of detections and the lowest overall detection probabilities (p = 0.52; Figure 2). The relationship of spotlight surveys to fox abundance was positive, but not significant (r^2^ = 0.21, P = 0.195; Figure 3C).

#### Trapping surveys

During the trapping surveys, we accumulated 2,640 capture nights and had 16 captures. Trapping had the second lowest detection probabilities (p = 0.59; Figure 2). The correlation between the indices from trapping surveys was significantly positive with kit fox abundance (r^2^ = 0.45, P = 0.017; Figure 3D).

#### Costs of surveys

The costs to perform our four surveys varied. Considering the costs of the initial supplies required (e.g., spotlights, scent-station tabs, traps), labor, and gas for the field trucks, the total cost to conduct one full survey along all 15 5-km transects during one biological season was $898 for the scat deposition transects, $940 for the spotlight survey, $2,406 for the trapping survey, and $2,760 for the scent-station survey. These costs would vary among study areas depending on differences in gasoline prices, labor costs, and the distance between transects.

## Discussion

Although once abundant on the DPG, kit fox abundance was low during this study. Capture success during trapping on the DPG was the second lowest reported in the literature at 0.017 (106 captures/6221 total trap nights) which is within the range of reported capture rates of 0.013 to 0.173 [41], [42]. This low capture rate may partially be due to our attempt to apply equal trapping effort across the entire study area, including areas known to be poor habitat for kit foxes and low numbers of foxes. During trapping at den sites, foxes were readily captured in one trap night and on most occasions we were able to capture the entire family group in a single night. One fox became ‘trap shy’ and the use of the tunnel trap [29] was successfully deployed to capture that individual for changing its radio-collar.

Low prey abundance and high intraguild predation by coyotes may be limiting kit fox density on the DPG [11], [20]. Since the 1950's, much of the DPG has been converted from native Great Basin Desert shrub communities to grasslands [11] which support reduced small mammal diversity and abundance [22]. Fox home range size was largely dependent on prey availability [41], [43]–[46]. In addition to this habitat change, the DPG has seen an increase in coyote abundance [11]. Predator-caused mortality was the highest cause of death for kit foxes during this study and coyotes have been shown to limit kit fox density [20], [22], [41], [45]. Mean home range size for kit foxes on the DPG was large (20.1 km^2^); similar to earlier study [22] with a mean home range size of 22.6 km^2^ for kit foxes on the DPG. Studies of kit foxes in other regions have reported much smaller home ranges between 4.6 km^2^ [46] and 13.7 km^2^ [47] with an average of 11.4 km^2^ [22], [43], [44], [46]–[50].

Scat deposition surveys consistently had the highest detection probability, were most closely related to fox abundance, and were relatively inexpensive to perform. Scat deposition transects required the greatest period of surveying (i.e., 14 days) which likely increased the chance of a sample being deposited and subsequently detected for this rare and widely dispersed species. Additionally, as a passive technique, scat deposition surveys do not require the target species to behave unnaturally (e.g., enter a trap or investigate a scent tab [15]). Our results corresponded with another study [6] finding scat deposition surveys to be the best method for detection of carnivores in the northeastern United States, and was similar to results for swift foxes in New Mexico [14].

Where misidentification and overlap with non-target species are a concern, training observers for accurate scat identification was critical [4], [14], [15]. But if multiple species are of concern, it would be possible to use this technique to efficiently identify multiple target species [19] with proper training. DNA analysis could also be used for verification of species and/or determining species abundance [4], [14], [17], [19], [51]. The use of scat detection dogs may increase detections rates [19], [51] and if the dog is trained to detect a particular species, it could assist in the proper identification of the target species [19]. During this study, misidentification of scat may be the cause of detections along transects without known foxes. The risk of misidentification was highest during the pup-rearing season when juvenile coyotes and red foxes have the highest overlap in scat diameter with kit foxes. Before conducting scat deposition surveys, one should be aware of seasonal defecation patterns and related concerns when estimating site occurrence or abundance [17].

Scent-station surveys had the second highest detection probability of the 4 techniques compared and the second highest correlation with the number of available foxes, but were the most expensive on all four techniques evaluated. Snow and freeze/thaw cycles during the winter months on the DPG could freeze the sifted sand, thereby diminishing any tracks left by a visiting fox. Also, periods of high winds were more common during the winter, thereby erasing any tracks. We found making a small imprint in the sand helpful in determining if a scent station was still operable. During the breeding season we completed 85% of survey nights; surveys were more reliably completed during the dispersal and pup-rearing season with operable station nights of 100% and 97%, respectively.

Similar to scat deposition transects, more than one target species may be detected at a scent station [36], although wariness of a species to sifted sand on the station should be considered [3]. This technique has the highest potential for observer bias and possible misidentification of tracks. Training of the observer at track identification is crucial to avoid misidentification, especially when there are multiple canids on the landscape. We found that 1–2 cm of sifted sand left the most discernible tracks. Our results were consistent with other studies showing a positive correlation of scent station detections to fox abundance [13]–[15], although one study reported fairly erratic results and suggested scent station surveys were only able to detect large changes in the population [13].

In this widely dispersed kit fox population, spotlight surveys were found ineffective at detecting fox presence and failed to detect a single fox during the dispersal season of 2011, although 18 known foxes were available. During 327 survey nights, we only detected fox presence 15 times. Spotlighting had the lowest detection probability and was not significantly correlated to fox abundance. Obstruction of view from vegetation and topography [4], [13], [15] are concerns when using this technique. In addition, highly mobile, wary species may actively evade detection [52]. This technique failed to detect kit foxes twice when foxes were known to be available for detection and was the weakest performing technique, similar to a study on swift foxes [15]. Spotlight surveys were found to be inefficient at detecting swift foxes in New Mexico [14].

The trapping survey was only slightly less correlated to fox abundance than the scent station survey, but had a much lower detection probability than both the scat deposition transects and scent station surveys. One of the main benefits from this technique was the ability to add ear tags to captured foxes to conduct capture-mark-recapture estimation of abundance [15]. Due to a low capture rate, low numbers of foxes, and high mortality from predation, we had very few recaptures and therefore could not perform mark-recapture abundance analyses. Because of concerns for the safety of trapped individuals (high summer temperatures) and possible effects on natal young, trapping surveys were not conducted during the pup-rearing season. Trapping posed the highest risk to the animal of all methods used as we did have 3 minor foot injuries and 4 mouth injuries. We recommend modifying the mesh size to a mesh size of 1–2 cm [15]. The effect of repeated trapping of foxes should also be considered [15]. We had a few animals become trap happy and were repeatedly captured, while one fox became trap shy and could only be recaptured using a tunnel trap [29].

For detecting kit foxes in a low density population we suggest using scat deposition transects during the breeding season. This method had both the highest detection probability and highest correlation to kit fox abundance. This method also resulted in lower costs and labor requirements, was resilient to weather, and entailed no risk to the study animals [15]. The breeding season was ideal for monitoring kit fox population size, as detections consisted of primarily the resident population and we had the highest detection probabilities during this season. In areas where overlap with other sympatric canids occurs, careful training of technicians may be required, but the risk of overlapping scat dimensions should be lowest during the breeding season as most sympatric canids are also fully grown by the subsequent breeding season.
